# Supplementary material for: A multistationary loop model of ALS unveils critical molecular interactions involving mitochondria and glucose metabolism
Source: PLoS One. 2020 Dec 17;15(12):e0244234. doi: 10.1371/journal.pone.0244234 (PMC7746301; doi:10.1371/journal.pone.0244234)
Supplement: Attachment — Submitted filename: Response to Reviewers.pdf [file pone.0244234.s001.pdf]

Author Response for manuscript PONE-D-20-26823

Modeling the pathogenesis of amyotrophic lateral sclerosis by a multistationary loop system involving mitochondria and glucose metabolism

Response to the Academic Editor

Thank you for submitting your manuscript to PLOS ONE. After careful consideration, we feel that it has merit but does not fully meet PLOS ONE's publication criteria as it currently stands. Therefore, we invite you to submit a revised version of the manuscript that addresses the points raised by our expert reviewer.

Dear Dr. Renping Zhou,

thank you for your positive assessment.

We are submitting a revised version that meets PLOS ONE's style requirements, and where all the points raised by the expert Reviewer have been thoroughly and carefully addressed.

We feel that the revision in line with the received comments has significantly improved the manuscript, and we are grateful to the Reviewer for his precious suggestions.

Kind regards,
Giulia Giordano

Journal Requirements:

When submitting your revision, we need you to address these additional requirements.
1. Please ensure that your manuscript meets PLOS ONE's style requirements, including those for file naming. The PLOS ONE style templates can be found at
https://journals.plos.org/plosone/s/file?id=wjVg/PLOSOne_formatting_sample_main_body.pdf
and
https://journals.plos.org/plosone/s/file?id=ba62/PLOSOne_formatting_sample_title_authors_affiliations.pdf

We have ensured that the manuscript meets PLOS ONE's style requirements as outlined in the provided templates.

<u>Response to Reviewer #1</u>

The paper titled "Modeling the pathogenesis of amyotrophic lateral sclerosis by a multistationary loop system involving mitochondria and glucose metabolism" presents a systems engineering/control theory analysis of three interlinked feedback loops involving the mitochondria of spinal motor neuron cells during Amyotrophic lateralsclerosis (ALS).

The manuscript presents timely and important work, novel and intriguing results.

We thank the Reviewer for the time he devoted to the review of our manuscript and for his very positive assessment.

However, the organization, writing and presentation make these results hard to understand and will limit the impact of this work. I believe it can be substantially improved, and I suggest the paper be accepted after a rewrite.

We have thoroughly and carefully addressed all the received comments, changing the organization, writing and presentation as the Reviewer suggested.
We believe that the revised manuscript is now indeed substantially improved.

1. Reorganization
- The Methods section is short, and it is confusing as to what the is trying to be said here.
- The definition of the model is fragmented and spread throughout the result section
- There is no clear division between how the model was constructed and the analysis of the model.
I suggest that the authors remove the "methods" section and replace it with a "model" section. Move to this new section (including subsections) all detail about the modeling methodology, how the model was developed, and details on the variable, interactions, and feedback loops. It is rare that I want more equations in a paper, but I feel that a few more might bring clarity to the model description.
In the "results" section, clearly describe each "computational experiment" (or analysis) you performed on the model, what the results of the analysis showed and what the implications are. I feel that more organization here will help show the power of your results.

We have completely reorganized the manuscript following the Reviewer's suggestions. The manuscript now includes a section ("The Model") that discusses in detail the modeling and methodology (both biological modeling of ALS pathogenic mechanism, and mathematical model and methods). This section has been further subdivided into three subsections, i.e. a Background subsection containing the assumptions for the development of the model, a subsection devoted to the definition of loops, and finally a subsection describing the mathematical approach. Thereafter, the Results section has been devoted to describing the mathematical analysis performed on the model, its results, and the ensuing implications.

2. Rewrite abstract
- The abstract is very dense, and does not read either clearly or informatively. I suggest rewrite/workshop/wordsmith the abstract. I think that a shorter, more sufficient abstract will increase the readability, and thus the impact of this paper.

As the Reviewer suggests, we have completely rewritten the abstract in a streamlined and more readable form. Most acronyms have been omitted in the effort of achieving better readability.

3. Consider changing the title
- I am always hesitant to recommend authors to change their title, however some revision here could help readers understand the content of the paper and increase the reach and impact of the publication. Informing the reader of what results you have in the title can be helpful.
One possible suggestion would be: "Modeling of ALS feedback loops elucidate critical molecular interactions".

We have replaced the title with a more informative one by following and rearranging the Reviewer’s suggestion.
